# Editorial: Navigating the microbial landscape: integrating mass spectrometry imaging and other multimodal imaging approaches for spatially resolved microbial studies

**DOI:** 10.3389/fcimb.2026.1887183

**Published:** 2026-06-09

**Authors:** Michael D. Tuck, Maureen Feucherolles, Peter D. E. M. Verhaert

**Affiliations:** 1 The Michael E. DeBakey Department of Surgery, Baylor College of Medicine, Houston, TX, United States; 2 Luxembourg Institute for Science and Technology, Belvaux, Luxembourg; 3 Department of Imaging and Pathology, University of Leuven, Leuven, Belgium; 4 School of Physiology and Morphology, University of the Witwatersrand, Johannesburg, South Africa; 5 ProteoFormiX BV, Vorselaar, Belgium

**Keywords:** mass spectrometry imaging (MSI), microbiology, microorganisms, multimodal imaging, spatial omics

The microbial world is more than a collection of species, but rather a spatially organized, metabolically dynamic ecosystem embedded within the tissue/environment it inhabits. Understanding microorganisms in the context of health and disease therefore demands tools capable of revealing not just identity, but location, activity, and molecular function, resolved within their native biological landscape. This Research Topic was established to bring together contributions that advance precisely this capacity, uniting mass spectrometry imaging (MSI) and complementary multimodal imaging approaches in service of spatially resolved microbial science.

The four articles assembled here span the breadth of this emerging field, from foundational method development to clinical application.

Nakagawa et al. address a technically challenging problem for gut microbiome research: the spatial visualization of short-chain fatty acids (SCFAs). These volatile microbial metabolites are difficult to detect in intact tissue. Through the introduction of a chemical derivatization strategy using TMPA and HATU reagents, carboxylic acids are converted into stable, ionizable quaternary amines, minimizing SCFA volatility in tissue. Subsequent MALDI-MSI detects butyrate and propionate directly within the murine cecum, co-localizing these metabolites with zones of high bacterial density confirmed by histological staining. Bacterial identity was further validated by MALDI proteotyping of cultured cecal isolates, creating a seamless link between microbial community and metabolic output within the same tissue.

Snyder et al. take a complementary optical approach, applying two-photon fluorescence lifetime imaging microscopy (2p-FLIM) to characterize, in a label-free manner, the metabolic perturbations induced by *Pseudomonas aeruginosa* infection across multiple biological models and states. By exploiting the intrinsic autofluorescence of NAD(P)H and employing phasor plot analysis, differences in metabolic state are resolved between bacterial strains, infected host cells, and cryo-sectioned lung tissue from a murine infection model. This reveals an upregulation of oxidative phosphorylation in the host during active infection and demonstrates near real-time quantitative metabolic mapping without the need for exogenous labels.

Extending the application of mass spectrometry to clinical diagnostics, Takei et al. report the first isolation of *Pseudoxanthomonas winnipegensis* from a blood culture in Japan and demonstrate that MALDI-MS, by identifying *m/z* features corresponding to ribosomal proteins L29 and L33 and the cold-shock protein CspA, can reliably differentiate five human-infecting *Pseudoxanthomonas* species that resist accurate identification by routine microbiological methods. This work highlights how molecular fingerprinting by mass spectrometry can resolve taxonomic ambiguity at the point of clinical need, with direct implications for the management of opportunistic infections caused by environmentally-derived organisms.

Situating these methodological and clinical advances within a broader biological framework, Meyers et al. provide a comprehensive review of the microbiome’s role in cancer metastasis, surveying how specific bacterial taxa, including *Fusobacterium nucleatum*, *Bacteroides fragilis*, and *Helicobacter pylori*, may influence four key steps of the metastatic cascade through direct invasion, toxin secretion, and immune modulation. Critically, the review argues that resolving the microbiome’s contribution to metastatic progression requires moving beyond compositional profiling toward spatially-resolved approaches. This includes MALDI-MSI, DESI-MSI, NanoSIMS, imaging mass cytometry, and spatial transcriptomics workflows that can map microbial communities, their metabolites, and their interactions with host cells directly within the tumor microenvironment.

Taken together, these four contributions articulate a unifying conviction at the heart of this Research Topic: that the biology of microorganisms is inseparable from their spatial and molecular context, and that imaging technologies, specifically MSI and complementary multimodal imaging, are the instruments that make that context achievable. Whether the objective is to map a metabolite to a bacterial niche in the gut, to capture the metabolic signature of a pathogen in a living host cell, to identify an organism from a clinical isolate, or to chart the microbiome’s influence across the stages of metastasis, each paper demonstrates that spatial resolution and molecular specificity are not technical luxuries but scientific necessities.

This Research Topic reflects a broader shift in approaching the *Biology of Microbes*: from cataloguing the microbiome “*who is present?*”, to understanding “*where everybody is*”, “*what they are doing*”, and, ultimately, “*how microbial activities shape the biological systems and environment they inhabit*”.

